# The structure of DNA methyltransferase DNMT3C reveals an activity-tuning mechanism for DNA methylation

**DOI:** 10.1016/j.jbc.2024.107633

**Published:** 2024-08-02

**Authors:** Nelli Khudaverdyan, Jiuwei Lu, Xinyi Chen, Genevieve Herle, Jikui Song

**Affiliations:** 1 Department of Biochemistry, University of California, Riverside, California, USA; 2 Biophysics Program, University of California, Riverside, California, USA

**Keywords:** *de novo* DNA methylation, DNMT3C, DNMT3A, DNMT3B, DNA methyltransferases, evolutionary covariation, CpG methylation, non-CpG methylation, substrate specificity

## Abstract

DNA methylation is one of the major epigenetic mechanisms crucial for gene regulation and genome stability. *De novo* DNA methyltransferase DNMT3C is required for silencing evolutionarily young transposons during mice spermatogenesis. Mutation of DNMT3C led to a sterility phenotype that cannot be rescued by its homologs DNMT3A and DNMT3B. However, the structural basis of DNMT3C-mediated DNA methylation remains unknown. Here, we report the structure and mechanism of DNMT3C-mediated DNA methylation. The DNMT3C methyltransferase domain recognizes CpG-containing DNA in a manner similar to that of DNMT3A and DNMT3B, in line with their high sequence similarity. However, two evolutionary covariation sites, C543 and E590, diversify the substrate interaction among DNMT3C, DNMT3A, and DNMT3B, resulting in distinct DNA methylation activity and specificity between DNMT3C, DNMT3A, and DNMT3B *in vitro*. In addition, our combined structural and biochemical analysis reveals that the disease-causing *rahu* mutation of DNMT3C compromises its oligomerization and DNA-binding activities, explaining the loss of DNA methylation activity caused by this mutation. This study provides a mechanistic insight into DNMT3C-mediated DNA methylation that complements DNMT3A- and DNMT3B-mediated DNA methylation in mice, unraveling a regulatory mechanism by which evolutionary conservation and diversification fine-tune the activity of *de novo* DNA methyltransferases.

DNA methylation is an evolutionarily conserved epigenetic mechanism that is important for silencing retrotransposons, which account for ∼40% of the mammalian genome (1, 2, 3). Retrotransposon silencing is essential for proper mammalian development, such as embryogenesis, gametogenesis, and sexual reproduction (4, 5). Failure to silence retrotransposons leads to genomic instability and consequently affects the fitness of subsequent generations (6). Germline is most vulnerable to this threat as some of the retrotransposons escape from the first wave of DNA methylation in early embryogenesis and must be silenced during the reprogramming of gametogenesis to avoid havoc in germ cells (6, 7).

The establishment of DNA methylation in mammals is mainly orchestrated by three paralogs of DNA methyltransferases (DNMTs): DNMT3A, DNMT3B, and DNMT3C, and an enzymatically inactive regulator DNMT3L (6, 8, 9, 10, 11, 12, 13, 14, 15). Among these, DNMT3C was evolutionarily derived from tandem duplication of DNMT3B but lacks a corresponding N-terminal Proline-Tryptophan-Tryptophan-Proline (PWWP) domain (6). The three members of the *de novo* DNA methyltransferase family share similar but distinctive functionalities during development, which is in part attributed to the diversification selection of their regulatory and catalytic domains (16, 17). It has been demonstrated that DNMT3A is required for methylating major satellite repeats and allele-specific imprinting during gametogenesis, and DNMT3B is important for methylation of minor satellite repeats during embryogenesis; knock out of either DNMT3A or DNMT3B in a mouse model led to prenatal fatality or death shortly after birth (18), indicating that both DNMTs are indispensable. In contrast, DNMT3C forms a methyltransferase complex with DNMT3L to exclusively methylate and silence evolutionally young yet transcriptionally active transposons, such as L1, EVKs, and IAPs, in the male germline (6, 7). DNMT3C mutation, such as the so-called *rahu* (recombination affected with hypogonadism from under-populated testes) mutation (E693G), which was identified through a forward genetics screen, led to meiotic failure defecting spermatogenesis, thereby affecting male sterility of mice (7).

Previous studies of DNMT3A and DNMT3B in the context of DNMT3A-DNMT3L and DNMT3B-DNMT3L complexes revealed a DNMT3L-DNMT3A/DNMT3B-DNMT3A/DNMT3B-DNMT3L tetrameric assembly, mediated by both the polar, homodimeric interface (RD interface) and the non-polar, heterodimeric interface (FF interface) (16, 19, 20, 21, 22, 23). The interaction between DNMT3A or DNMT3B methyltransferase (MTase) domain and DNA substrates is mediated by a loop from the target recognition domain (TRD loop), an α-helix at the RD interface (RD helix), and the catalytic loop (16, 19, 22, 23). Of note, the catalytic loop and the TRD loop coordinately govern the CpG specificity of DNMT3A/DNMT3B. An intramolecular hydrogen bond in the catalytic loop, present between DNMT3B N656 and R661 but not between the corresponding sites (I715 and R720) of DNMT3A, gives rise to a higher methylation activity but a lower CpG specificity for DNMT3B than DNMT3A (16). Introducing DNMT3A-converting mutation to DNMT3B or DNMT3B-converting mutations to DNMT3A on these sites led to a switch of the activity and substrate specificity between the two enzymes (16). In addition, DNMT3A and DNMT3B show different context-dependent interactions with the +1 CpG-flanking site, resulting in their distinct flanking sequence preference (16). On the other hand, how DNMT3C, the MTase domain of which bears ∼92% and ∼77% sequence identity with the corresponding regions of DNMT3B and DNMT3A, respectively (Fig. S1), attains its substrate recognition and specificity remains unknown.

To provide the molecular basis for DNMT3C-mediated DNA methylation, we determined the crystal structure of the DNMT3C MTase domain in complex with DNMT3L and CpG-containing DNA. Whereas the substrate-recognition mechanism of DNMT3C resembles that of DNMT3A and DNMT3B, the DNA methylation activity of DNMT3C is restricted by the unique presence of a glutamate (E590) in the DNA-contact sites. Furthermore. our combined sequence, structural, and biochemical analysis reveals that residues C543 and E590 constitute two coevolving sites that impact the catalytic-loop conformation of DNMT3C, DNMT3A, and DNMT3B differentially, which consequently leads to distinct DNA methylation activity and specificity between DNMT3C, DNMT3A and DNMT3B. In addition, our biochemical and enzymatic analysis of the *rahu* mutant of DNMT3C, residing near to the RD interface, reveals a defect in protein oligomerization and DNA-binding activity, thereby explaining the loss of DNA methylation activity caused by this mutation. Together, this study reveals how evolutionary conservation and diversification fine-tune the enzymatic activity and specificity of *de novo* DNA methyltransferases, which may contribute to their distinct functionalities.

## Results

### Structural overview of the DNMT3C-DNMT3L-CpG DNA complex

**Figure 1:**
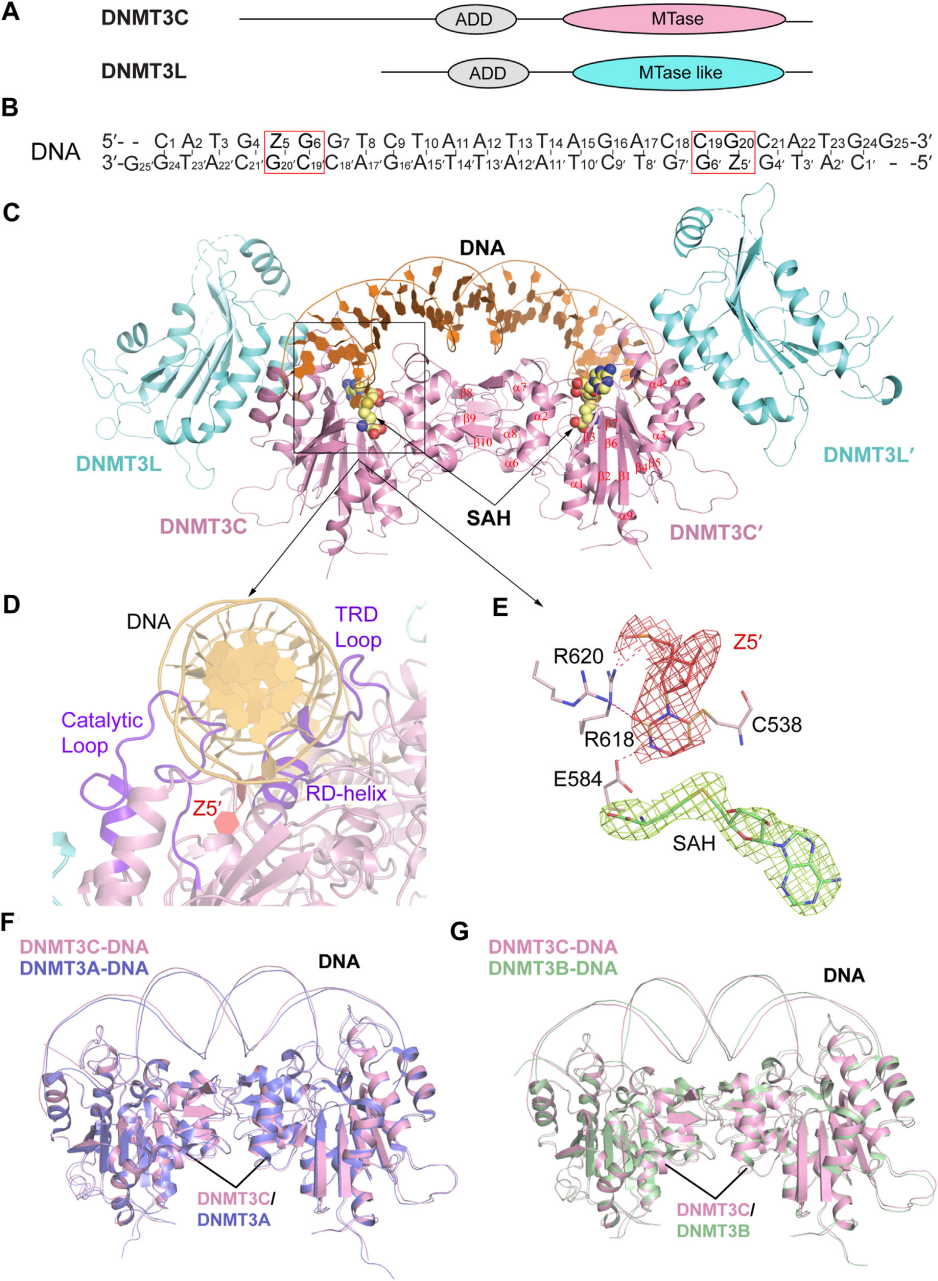
**Structure of the DNMT3C-DNMT3L tetramer bound to a CpG DNA.***A*, domain architecture of DNMT3C and DNMT3L. *B*, the DNA sequence used for structural study. Z denotes zebularine, a cytosine analog. *C*, structure of DNMT3C-DNMT3L bound to CpG DNA and SAH (sphere representation). *D*, close-up view of DNA-interacting RD helix, TRD loop, and Catalytic loop. *E*, close-up view of the catalytic site of DNMT3C bound to ZpG/CpG DNA. Fo-Fc omit maps for the flipped zebularine (Z5′) and the SAH molecule at 2σ contour level were shown as *salmon* and *green* mesh, respectively. The hydrogen bonds are shown as dashed lines. *F*, structural overlay of the DNMT3C-DNMT3L-DNA and DNMT3A-DNMT3L-DNA complex (PDB 6W8B). *G*, structural overlay of the DNMT3C-DNMT3L-DNA and DNMT3B-DNMT3L-DNA complex (PDB 6KDA).

The complex of DNMT3C with DNMT3L and CpG DNA was prepared as previously described for the DNMT3A-DNMT3L-DNA and DNMT3B-DNMT3L-DNA complexes (16, 19, 22, 23). For the purpose of crystallization, we focused on the mouse DNMT3C MTase domain and hDNMT3L C-terminal domain for structural determination (Fig. 1*A*). Note that the C-terminal domains of hDNMT3L or mDNMT3L, which do not engage in DNA interaction (16, 19, 22, 23), share similar oligomerization-interface residues (Fig. S2*A*). Accordingly, a comparison of the *in vitro* DNA methylation activity of the DNMT3C-mDNMT3L and DNMT3C-hDNMT3L on CpG-, CpA-, and CpT-containing DNAs revealed a similar substrate specificity on CpG- and non-CpG-containing DNAs (Fig. S2, *B*–*D*).

The DNA substrate is a 25-mer, self-complementary DNA containing a zebularine followed by guanine (ZpG) site near one end and a CpG site near the other end. Annealing of this DNA results in a duplex with two ZpG/CpG sites in a spacing of 14 base pairs (Fig. 1*B*). As established previously (16, 19, 23, 24, 25), the replacement of the target cytosine with a zebularine permits the formation of a stable DNMT3C-DNA covalent adduct. The crystal structure of the DNMT3C-DNMT3L-DNA complex bound to *S*-adenosyl-homocysteine (SAH), the by-product of cofactor S-adenosyl-methionine (SAM), was solved at 3.2-Å resolution (Fig. 1*C* and Table S1).

The structure of the DNMT3C-DNMT3L-DNA complex reveals a heterotetrameric assembly, organized in a DNMT3L-DNMT3C-DNMT3C-DNMT3L fashion reminiscent of those previously observed for the DNMT3A-DNMT3L-DNA and DNMT3B-DNMT3L-DNA complexes (Fig. 1*C*) (16, 19, 22, 23). As previously observed for the DNMT3A and DNMT3B complexes, the interaction between DNMT3C and DNA is mainly mediated by three distinct regions: TRD loop (residues 660–675) that interacts with DNA major groove, the catalytic loop (residues 535–559) that interacts with the DNA minor groove, and the RD helix that interacts with the backbone of the central DNA segment (Figs. 1*D* and S3, *A* and *B*). At the active site of each DNMT3C subunit, the zebularine (Z5/Z5′) on the target strand is flipped out of DNA helix and covalently bound to catalytic cysteine C538 of DNMT3C (Fig. 1*E*). In addition, the base of Z5/Z5′ is stabilized *via* hydrogen-bonding interactions with residues E584, R618, and R620 in the active site (Fig. 1*E*).

It is worth mentioning that in this study, the DNMT3C-bound DNA contains a guanine (G7) at the +1 ZpG-flanking site (Fig. 1*B*). Structural comparison of the DNMT3C-DNMT3L-CGG DNA complex with human DNMT3B-DNMT3L-CGG DNA complex (PDB 6KDA) and human DNMT3A-DNMT3L-CGA DNA (PDB 6W8B) reveals high structural similarity, with root-mean-square deviation (RMSD) of 0.49 Å and 0.48 Å over 548 and 545 aligned Cα atoms, respectively (Fig. 1, *F* and *G*), in line with the high sequence identity between the MTase domain of DNMT3C and that of DNMT3B or DNMT3A (Fig. S1).

### Residue E590 restricts the DNA methylation activity of DNMT3C

**Figure 2:**
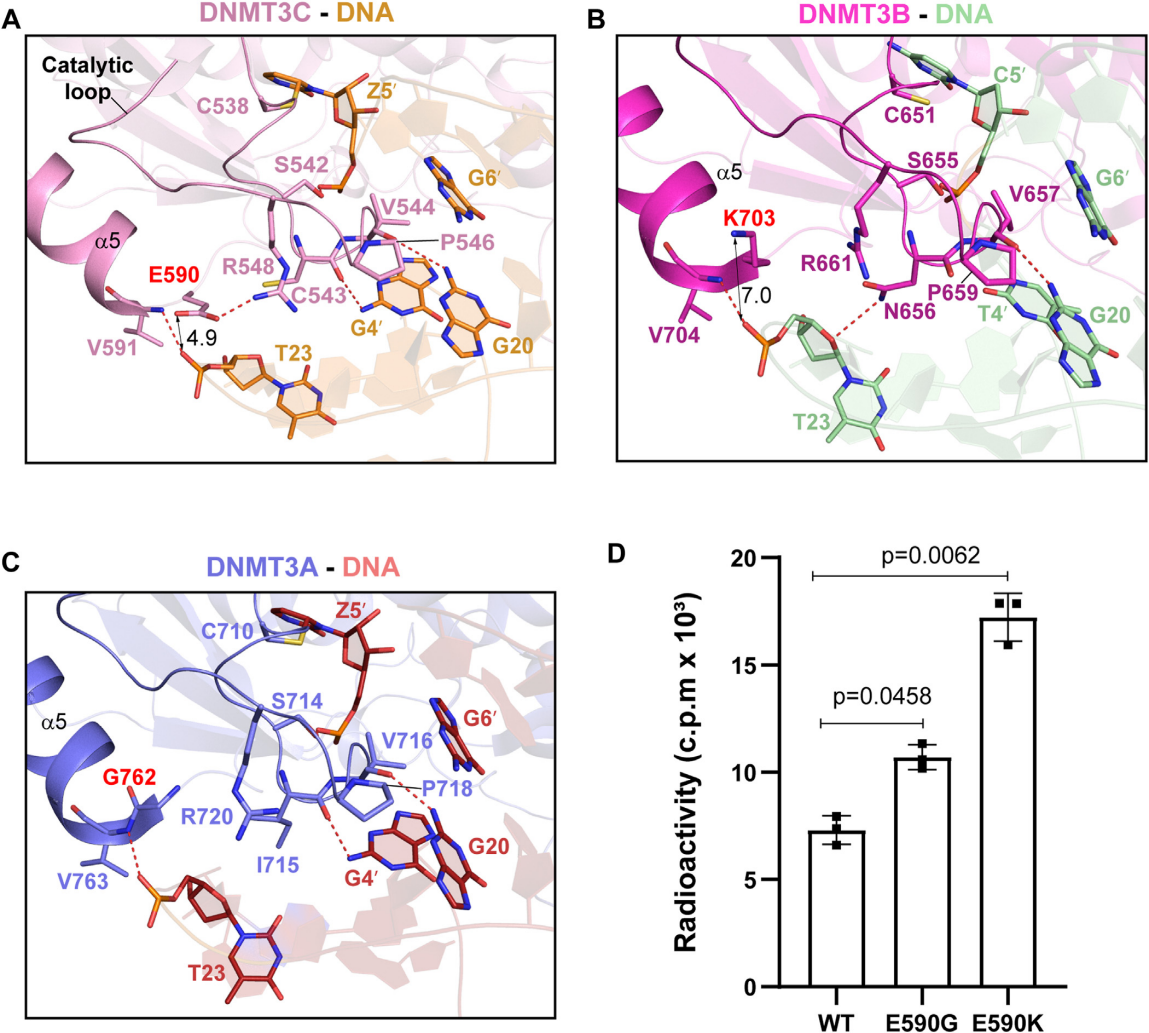
**Amino acid variation at the DNMT3C E590-corresponding site leads to distinct DNA interaction between DNMT3C, DNMT3B, and DNMT3A.***A*, close-up view of the interactions between DNMT3C E590, the catalytic loop of DNMT3C, and DNA. *B*, close-up view of the interactions between DNMT3C E590-corresponding site, DNMT3B K703, the catalytic loop of DNMT3B, and DNA. *C*, close-up view of the interactions between DNMT3C E590-corresponding site, DNMT3A G762, the catalytic loop of DNMT3A, and DNA. *D*, *in vitro* DNA methylation activities of DNMT3C, WT or mutant, on CpG DNA. Data are mean ± s.d. (n = 3 biological replicates). Statistical analysis for WT DNMT3C vs mutants used a two-tailed Student’s *t* test.

Toward the DNA minor groove, the DNMT3C MTase domain interacts with both DNA strands *via* the catalytic loop, involving residues C538, S542, C543, V544, and P546 (Figs. 2*A* and S3*B*). In addition to the covalent bonding between DNMT3C C538 and Z5′, DNMT3C V544 extends its side chain to occupy the DNA cavity vacated by Z5′, stabilizing the unpaired Gua20 *via* a hydrogen bond between the backbone carbonyl group of V544 and the N2 atom of the Gua20 (Fig. 2*A*). Furthermore, the backbone carbonyl of C543 receives a hydrogen bond from the N2 atom of Gua4; the sidechain hydroxyl group of S542 donates a hydrogen bond to the backbone phosphate of Z5′; and the prolyl ring of P546 interacts with the sugar/base moieties of Gua6′/Gua20 *via* van der Waals contacts (Fig. 2*A*). In addition to the catalytic loop, the α5-helix located at the DNMT3C-DNMT3L interface further stabilizes the DNMT3C-DNA association *via* a backbone hydrogen bond between DNMT3C V591 and Thy23 (Fig. 2*A*).

Structural comparison of the DNMT3C-DNMT3L-DNA complex with DNMT3A-DNMT3L-DNA and DNMT3B-DNMT3L-DNA complexes reveal subtle but notable differences in the minor-groove interaction (Fig. 2, *A*–*C*). The most striking difference lies in the N-terminus of α5-helix, where DNMT3C E590 is replaced by a glycine (G762) in DNMT3A but a lysine (K703) in DNMT3B (Fig. 2, *A*–*C*). In the DNMT3C-DNMT3L-DNA complex, the side chain of residue E590 forms a salt-bridge with catalytic-loop residue R548, which places E590 in proximity with the backbone of Thy23 for electrostatic repulsion (Fig. 2*A*). In contrast, the charge reversal for the corresponding site (K703) in DNMT3B leads to an electrostatic attraction with the DNA backbone in the DNMT3B-DNMT3L-DNA complex, thereby contributing to the substrate binding of DNMT3B (Fig. 2*B*). Different from DNMT3C and DNMT3B, the corresponding residue G762 in the DNMT3A-DNMT3L-DNA does not engage in appreciable DNA interaction (Fig. 2*C*). Sequence analysis of DNMT3C across various species indicates that residue E590 is highly conserved throughout evolution (Fig. S4), implying an important role of this residue in DNMT3C function.

To examine the effect of amino acid variation on the DNMT3C E590-corresponding site between DNMT3C, DNMT3A, and DNMT3B, we compared the *in vitro* DNA methylation activities of DNMT3C, DNMT3A, DNMT3B, as well as DNMT3A-converting mutant E590G and DNMT3B-converting mutant E590K of DNMT3C, on a 36-mer CpG-containing DNA duplex. Under the experimental condition, we observed a differential DNA methylation activity for DNMT3C, DNMT3A, and DNMT3B, with DNMT3B possessing a much higher activity than DNMT3C and DNMT3A (Fig. S5*A*). On the other hand, introducing the DNMT3A-converting E590G mutation and DNMT3B-converting E590K mutation to DNMT3C both substantially increase its DNA methylation efficiency, with the E590K mutation exhibiting a stronger effect that matches the relatively higher activity of DNMT3B (Fig. 2*D*). These data support the structural observations that the amino acid variation at the DNMT3C E590-corresponding sites influences the DNA interaction of DNMT3C, DNMT3A, and DNMT3B differentially, which contributes to the different DNA methylation activities among the DNMT3 family of 10.13039/100026054DNA methyltransferases.

### Residues C543 and E590 coordinately modulate the DNA methylation activity and specificity of DNMT3C

**Figure 3:**
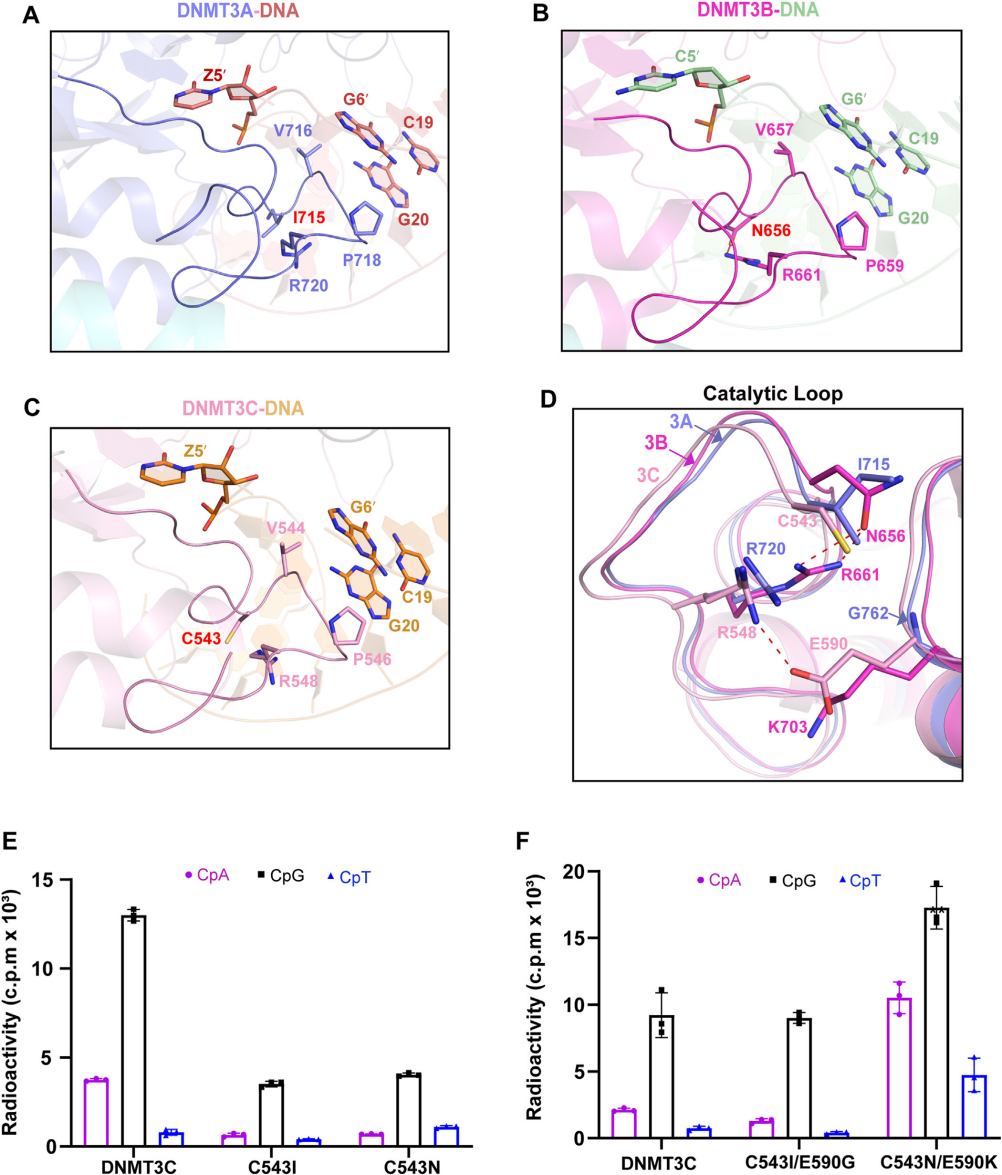
**An intramolecular interaction of the catalytic loop controls the CpG specificities of DNMT3A, DNMT3B, and DNMT3C.***A–C*, structural comparison of the DNMT3C-DNA, DNMT3A–DNA (PDB 6W8B), and DNMT3B–DNA (PDB 6KDA) complexes, highlighting distinct intramolecular interactions within the catalytic loops of DNMT3A (*A*), DNMT3B (*B*) and DNMT3C (*C*). *D*, close-up view of the overlaid catalytic loops of DNMT3A, DNMT3B, and DNMT3C, the DNMT3C C543-, R548-, and E590-corresponding sites shown in stick representation. The hydrogen-bonding interactions are shown as dashed lines. *E* and *F*, *in vitro* DNA methylation activities of DNMT3C, WT or that harboring single (*E*) or double (*F*) mutations, on CpA, CpG, and CpT DNAs. Data are mean ± s.d. (n = 3 biological replicates).

Sequence comparison of the catalytic loops of DNMT3A, DNMT3B, and DNMT3C reveals three sites that undergo amino acid variations, which correspond to DNMT3C C543, V547, and F552 (Figs. S1 and S4). Among these, the DNMT3C C543-corresponding site is involved in DNA interaction (Fig. 2, *A*–*C*). Along the line, previous studies have revealed that the DNA-bound DNMT3A and DNMT3B proteins exhibit a similar but distinct catalytic-loop conformation: the catalytic loop of DNMT3B harbors a hydrogen bond between residues N656 and R661, whereas no such interaction was observed for the corresponding pair (I715 and R720) in DNMT3A (Figs. 3, *A* and *B* and S5*B*) (16, 19, 22, 23). Such a conformational difference is associated with a higher DNA methylation efficiency but a reduced CpG specificity for DNMT3B than DNMT3A, likely caused by differential DNA contacts by the catalytic-loop residues (*e.g.* DNMT3A V716 and P718-corresponding residues) (Fig. 3, *A* and *B*) (16).

Given the fact that the corresponding pair C543/R548 in DNMT3C, as with that in DNMT3A, does not form a hydrogen bond within the pair (Fig. 3, *C* and *D*), we set out to determine whether the amino acid variation on the DNMT3C C543-corresponding site influences the DNA methylation activity and substrate specificity of DNMT3C. Toward this, we first compared *in vitro* DNA methylation activities between WT DNMT3C, DNMT3A, and DNMT3B on CpG-, CpA- and CpT-containing DNA substrates. Among the three *de novo* DNA methyltransferases, DNMT3B methylates CpG and non-CpG DNAs both in the highest efficiency under the experimental condition (Fig. S5*A*). As previously observed (16), whereas DNMT3B shows a substrate preference in the order of CpG > CpA > CpT (Fig. S5*A*), the relatively high methylation activity of DNMT3B on the non-CpG DNA results in a CpG specificity (relative methylation efficiency of ∼1.9-fold for CpG/CpA and ∼7.2-fold for CpG/CpT) lower than that of DNMT3A (relative methylation efficiency of 30.0-fold for CpG/CpA and 45.2-fold for CpG/CpT) (Fig. S5*A*). Interestingly, whereas DNMT3C methylates the CpG DNA in an efficiency similar to that of DNMT3A, it shows a modestly higher methylation efficiency on non-CpG DNAs, especially CpA DNA (Fig. S5*A*), which gives rise to a CpG specificity (relative methylation efficiency of ∼3.9-fold CpG/CpA and ∼14.3-fold CpG/CpT) closer to that of DNMT3B. Next, we mutated DNMT3C C543 into isoleucine (C543I) or asparagine (C543N), mimicking the corresponding sites in DNMT3A and DNMT3B, respectively. Our *in vitro* DNA methylation analysis revealed that introducing the C543I and C543N mutations to DNMT3C each led to a notable decrease in DNA methylation efficiency on CpA- and CpG-containing DNAs (Fig. 3*E*). This observation suggests that introducing the C543I or C543N mutation alone to DNMT3C is insufficient to convert its DNA methylation activity and specificity toward those of DNMT3A and DNMT3B, respectively.

As described earlier, the DNA-bound DNMT3A, DNMT3B, and DNMT3C also show distinct interactions between the DNMT3C E590-and R548-corresponding sites, with an intramolecular hydrogen bond formed between residues R548 and E590 of DNMT3C but not for the corresponding pairs in DNMT3A or DNMT3B (Fig. 3, *A*–*D*). These observations therefore establish a link between DNMT3C C543-, R548-and E590-corresponding sites, prompting us to investigate a potential interplay between the DNMT3C E590-corresponding site and the catalytic loop. Indeed, introducing the DNMT3B-converting C543N/E590K double mutation to DNMT3C greatly increased its methylation efficiencies on CpG, CpG and CpT, resulting in a CpG specificity (relative methylation efficiency of ∼1.6-fold for CpG/CpA and ∼3.6-fold for CpG/CpT) similar to that of DNMT3B (Figs. 3*F* *vs.* S5*A*). Likewise, introducing the DNMT3A-converting C543I/E590G double mutation to DNMT3C shifted both its DNA methylation efficiencies and CpG specificity (relative methylation efficiency of ∼7.0-fold for CpG/CpA and ∼21.9-fold for CpG/CpT) toward those of DNMT3A (Figs. 3*F* *vs*. S5*A*). Note that the relative CpG specificity for the C543I/E590G-mutated DNMT3C remains lower than that of DNMT3A, likely owing to the differential DNA contacts by their respective TRD loops (See details below). Next, we performed steady-state kinetic assays to compare the enzymatic kinetics of DNMT3C and its DNMT3A- or DNMT3B-converting mutants on CpG DNA (Fig. S6). In the context of DNMT3C-DNMT3L complex, a *k*_cat_ of 0.025 min^-1^ was determined for WT DNMT3C (Fig. S6*F*). Consistent with the single time-point measurements described above, introducing the single mutations C543N and C543I decreased the *k*_cat_ of DNMT3C to 0.014 min^-1^ and 0.011 min^-1^, respectively, whereas introducing the double mutation C543N/E590K and C543I/E590G increased the *k*_cat_ to 0.074 min^-1^ and 0.026 min^-1^, respectively (Fig. S6*F*). Together, these observations suggest that the DNMT3C C543- and E590-corresponding sites coordinately modulate the DNA methylation activities and specificities of DNMT3A, DNMT3B, and DNMT3C.

### Evolutionary covariation between DNMT3C C543- and E590-corresponding sites

Next, we performed sequence analysis between the subfamilies of DNMT3A, DNMT3B, and DNMT3C, focusing on the DNA-binding sites of the catalytic core. As expected from their close evolutionary link, the catalytic loop-surrounding region is highly conserved among DNMT3A, DNMT3B, and DNMT3C (Fig. S4). Nevertheless, several amino acid variations were mapped onto the catalytic loop and its subsequent helix (α4), as well as the spatially proximate α5-helix (Fig. S4). Among these are the DNMT3C C543- and E590-corresponding sites, which are strictly conserved within each DNMT3A, DNMT3B, or DNMT3C subfamily but distinct between subfamilies (Fig. S4). This observation, together with the fact that the DNMT3C C543- and E590-corresponding sites coordinately modulate the DNA methylation activity and specificity of DNMT3C, DNMT3A, and DNMT3B (Fig. S4), suggests that DNMT3C C543- and E590-corresponding sites have coevolved to confer distinct enzymatic activities and specificities of DNMT3C, DNMT3A and DNMT3B. How such an activity-tuning mechanism influences the respective DNA methylation by DNMT3A, DNMT3B, and DNMT3C *in vivo* awaits further investigation.

### DNMT3C TRD loop- and RD helix-mediated DNA interactions

**Figure 4:**
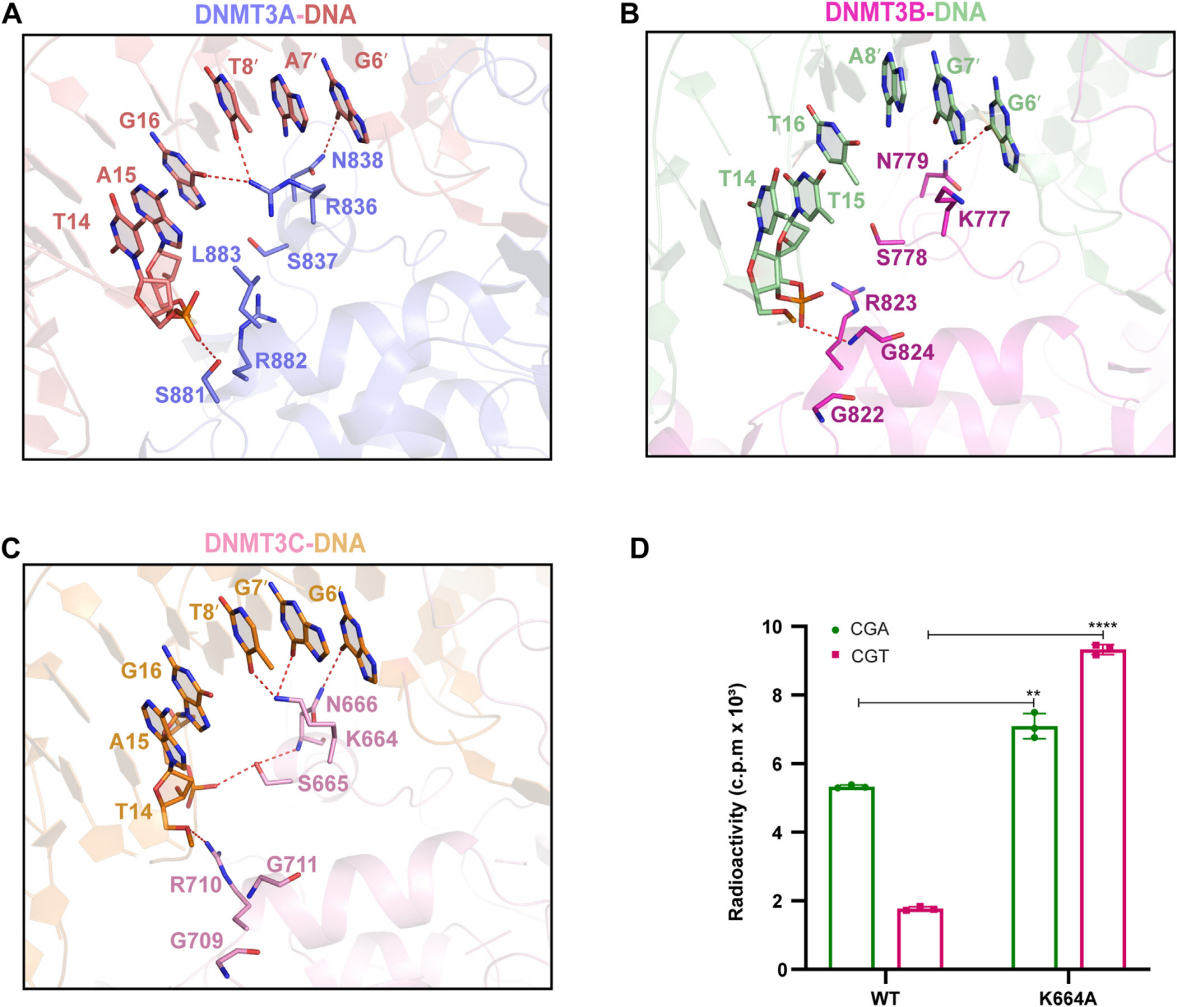
**The TRD loop- and RD helix-mediated DNA interactions of DNMT3C resemble those of DNMT3B.** Structural overlay of the TRD loop of DNMT3C-DNA, DNMT3A-DNA, and DNMT3B-DNA complexes, with DNA-interacting residues and the corresponding DNAs shown in stick representation. *A–C*, close-up view of the DNA interaction involving the TRD loop and RD helix of DNMT3A (*A*), DNMT3B (*B*), and DNMT3C (*C*). Hydrogen-bonding interactions are shown as dashed lines. *D*, *in vitro* DNA methylation activity of DNMT3C, WT or K664A, on CGA and CGT DNAs. Data are mean ± s.d. (n = 3 biological replicates). The two-tailed Student’s *t* test was used for statistical analysis for WT DNMT3C vs K664A. ∗∗*p* < 0.01. ∗∗∗∗*p* < 0.0001.

It has been established that the TRD loops of DNMT3A and DNMT3B modulate their respective CpG specificity as well as flanking sequence preference through interacting with the CpG and the +1-flanking sites (16, 19, 22, 23). Notably, DNMT3A and DNMT3B differ in the DNMT3B K777-corresponding site, which is an arginine in DNMT3A (R836) (Fig. 4, *A* and *B*). Previous studies from others and we have further demonstrated that DNMT3A and DNMT3B involve different context-dependent CpG interactions: in the context of the CGA or CGG motif, both DNMT3A R836 and DNMT3B K777 interact with the CpG-flanking sites (Fig. 4, *A* and *B*) (16, 19, 22, 23); in the context of CGT or CGC motif, DNMT3A R886 engages in a hydrogen-bonding interaction with CpG guanine, while DNMT3B K777 remains engaged with the +1/+2-flanking sites (16, 19, 22, 23). Such a difference in context-dependent CpG recognition between DNMT3A and DNMT3B coincides with the fact that DNMT3A prefers a pyrimidine over purine at the +1-flanking site, while DNMT3B prefers a purine over pyrimidine on the +1-flanking site (16, 26, 27).

DNMT3C contains a TRD loop that shares an identical sequence with the corresponding region of DNMT3B (Fig. S1). Consistently, structural comparison of the DNMT3C-CGG DNA and DNMT3B-CGG DNA interactions reveals a similar conformation for the TRD loop (Fig. 4, *B* and *C*). Of note, DNMT3C N666 interacts with CpG guanine (Gua6) *via* a hydrogen-bonding interaction, as with the corresponding DNMT3B N779 in the CGG complex and the corresponding DNMT3A N838 in the CGA complexes (Fig. 4, *A*–*C*). Furthermore, DNMT3C K664 engages hydrogen-bonding and/or van der Waals contacts with the +1- and +2-flanking sites in a manner similar to the corresponding DNMT3A R836 in the CGA complex and DNMT3B K777 in the CGG complex (Fig. 4, *A*–*C*), reinforcing the notion that the corresponding residue mediates the recognition of CpG-flanking sequences. Nevertheless, in comparison with the DNMT3B K777, DNMT3C K664 appears closer to the +1- and +2-flanking sites for hydrogen-bonding interactions, presumably due to the fact that the DNA molecules in the two complexes used for structural study differ in the +2 site (A8′ in the DNMT3B-CGG vs T8′ in the DNMT3C-CGG; Fig. 4, *B* and *C*). To test the role of residue K664 on the flanking sequence preference of DNMT3C, we performed *in vitro* DNA methylation assays for DNMT3C, WT and K664A mutant, on (CGA)_12_ and (CGT)_12_ DNA duplexes, in which the CpG sites on the complementary strands are methylated. WT DNMT3C shows a higher methylation efficiency on CGA DNA than on CGT DNA (Fig. 4*D*). However, introducing the K664A mutation not only increased the overall DNA methylation efficiency of DNMT3C on both CGT and CGA DNAs but also shifted the relative methylation flanking preference from CGA to CGT (Fig. 4*D*). These observations recapitulate what was previously observed for the corresponding DNMT3B K777A mutation (16), confirming that the TRD loops of DNMT3C and DNMT3B share conserved CpG-recognition mechanisms. A detailed comparison of the flanking sequence preference between DNMT3C and DNMT3B awaits further investigation.

It is worth noting that the RD helix of DNMT3C also shares a similar DNA interaction mode with that of DNMT3B, both involving an arginine (R710 in DNMT3C and R823 in DNMT3B) for an interaction with the DNA backbone (Fig. 4, *B* and *C*). In contrast, the corresponding region of DNMT3A engages in more extensive interactions, involving residues S881, R882, L883, and R887, for the DNA contact (Fig. 4*A*). The functional consequence of the amino acid variation on the RD helix between DNMT3s remains to be determined.

### The effect of *rahu* mutation on DNMT3C activity

**Figure 5:**
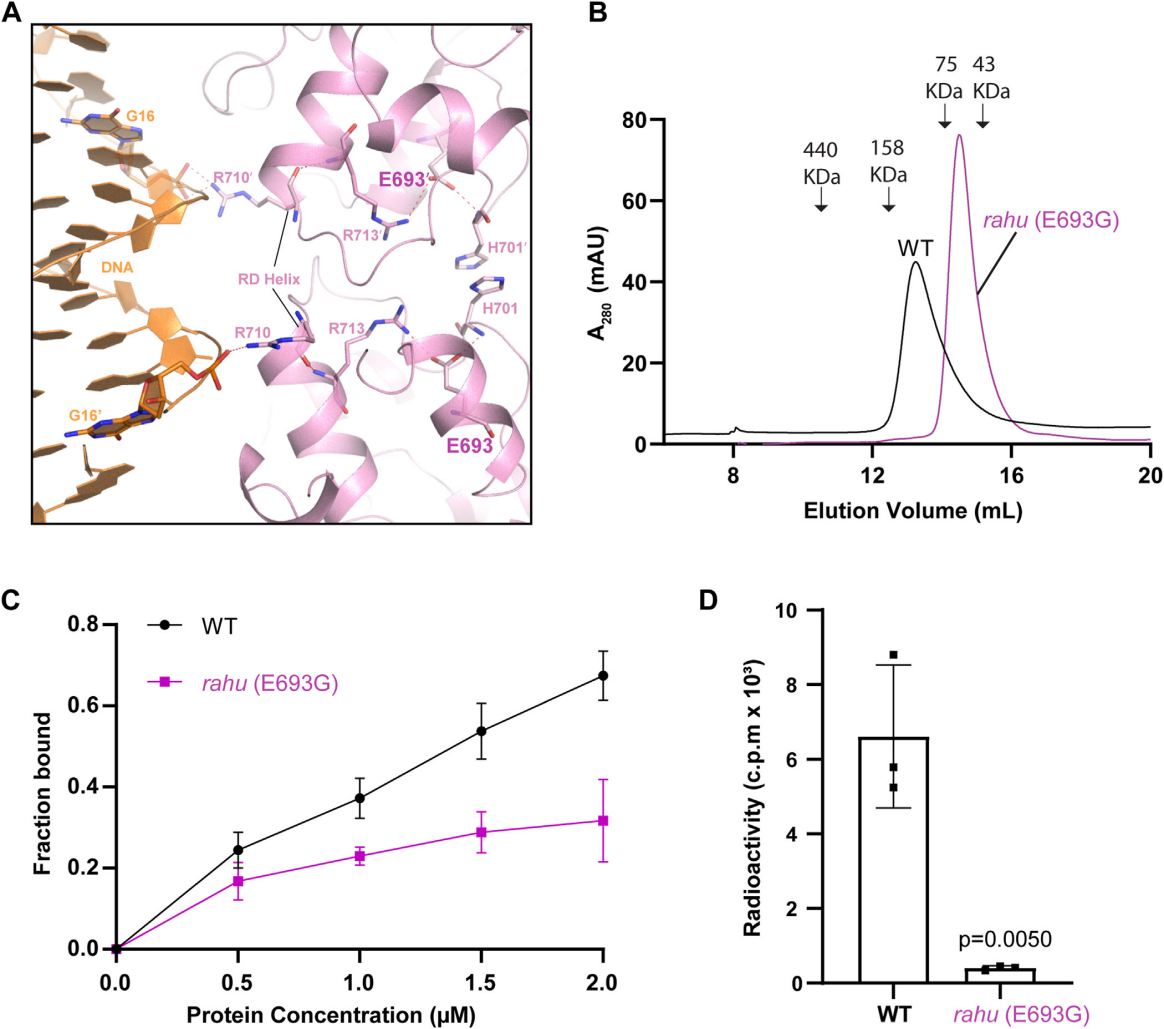
**Structural and biochemical analyses of DNMT3C *rahu* mutant.***A*, close-up view of the DNMT3C E693-engaged interactions at the RD interface of DNMT3C-DNA complex. Hydrogen-bonding interactions are shown as dashed lines. *B*, size-exclusion chromatography analysis of WT and *rahu* mutant DNMT3C. The elution volumes for the select protein markers are indicated by arrows. Note that the molecular weight for tetrameric and dimeric DNMT3C-DNMT3L are ∼134 kD and ∼67 kD, respectively. *C*, the ratio of DNA-bound WT or mutant DNMT3C-DNMT3L, determined by the EMSA analysis, as a function of protein concentration. Data are mean ± s.d. (n = 3 biological replicates). *D*, *in vitro* DNA methylation analysis of DNMT3C, WT or *rahu* mutant, on CpG DNA. Data are mean ± s.d. (n = 3 biological replicates). Statistical analysis for WT DNMT3C vs rahu used two-tailed Student’s *t* test.

A previous study has demonstrated that DNMT3C E693G, the so-called *rahu* mutation, is responsible for the male sterility phenotype in mice (7). Close inspection of the DNMT3C-DNMT3L-DNA complex reveals that residue E693 interacts with the RD-interface residues, such as H701 and R713, *via* hydrogen-bonding interactions. Conceivably, the hydrogen-bonding interaction between DNMT3C E693 and H701 may help stabilize the homodimerization of DNMT3C (Fig. 5*A*). In addition, the hydrogen-bonding interaction between residues E693 and R713 may stabilize the DNA-contacting helix at the RD interface (where residue R710 is located) (Fig. 5*A*), thereby contributing to the DNA-binding activity of DNMT3C. In support of these notions, our size-exclusion chromatography analysis reveals that the *rahu* mutation shifts the elution of DNMT3C-DNMT3L from a volume that corresponds to the heterotetrametric assembly to a dimeric assembly (Fig. 5*B*), suggesting disruption of the tetrameric assembly of DNMT3C-DNMT3L by the *rahu* mutation. Next, we performed electrophoretic mobility shift assay (EMSA) for DNMT3C, WT and *rahu* mutant. Increasing concentration of WT DNMT3C gradually decreased the intensity of the free DNA band, indicative of the complex formation (Figs. 5*C* and S7). In contrast, titration of DNA with the *rahu* mutant of DNMT3C did not lead to an appreciable shift of the DNA band (Figs. 5*C* and S7). These data therefore support that the *rahu* mutation led to disruption of homodimerization as well as reduced DNA-binding activity of DNMT3C. Finally, our *in vitro* DNA methylation analysis reveals that the *rahu* mutation led to largely abolished DNA methylation activity of DNMT3C (Fig. 5*D*), consistent with a previous observation that the *rahu* mutation caused DNA hypomethylation in cells (7). Together, these data establish that the *rahu* mutation compromised DNMT3C-mediated DNA methylation *via* impairing its oligomerization and DNA-binding activity.

## Discussion

DNA methylation plays a critical role in transposon silencing and chromatin compartmentalization during development. Dysregulation of DNA methylation leads to various developmental defects and diseases. The recent identification of DNMT3C, which specifically methylates promoters of young transposons in mouse sperm (6, 7), provides a mechanism for silencing the evolutionarily young transposons (*e.g.* EVRKs) that are enriched in the mouse genome (3), thereby shedding light on how mammalian DNA methyltransferases adapt to the genome complexities of rodents through functional partitioning. DNMT3A, DNMT3B and DNMT3C co-exist in mouse male germ cells (6, 28). Compared with closely related DNMT3B, the lack of an N-terminal histone reader PWWP domain in DNMT3C may contribute to its distinct chromatin targeting activity. On the other hand, how the DNA methylation activity of DNMT3C is regulated to maintain a proper balance of DNA methylation across the genome remains unclear. This study, through combined structural and biochemical analysis, provides a mechanistic insight into DNMT3C-mediated *de novo* DNA methylation and the functional impact of its disease mutation.

First, this study reveals that DNMT3C interacts with substrate DNA in a manner similar to but distinct from that of DNMT3A and DNMT3B. On the one hand, the interaction between the TRD loop of DNMT3C and DNA resembles the DNMT3B-DNA interaction, both recognizing the CpG guanine *via* an asparagine (DNMT3C N666 or DNMT3B N779) and the CpG-flanking sites *via* a lysine (DNMT3C K664 or DNMT3B K777). On the other hand, substantial diversification among the three *de novo* DNA methyltransferases was observed for the interaction between their Rossmann-fold catalytic core and DNA. Of note, DNMT3C contains a glutamate at site 590, rather than a lysine (K703) in DNMT3B or a glycine (G762) in DNMT3A. The presence of E590 results in electrostatic repulsion between DNMT3C and DNA substrate, thereby restricting the DNA methylation activity of DNMT3C that may help avoid genome-wide DNA hypermethylation.

Evolutionary covariation of two functional sites is recurrently observed for enzymes, which leads to fine-tuning of the enzymatic activity, thereby adapting them to distinct environments encountered by various species (29, 30). This study shows that the replacement of DNMT3C C543 by the corresponding residues in DNMT3A and DNMT3B led to a significant decrease in the DNA methylation activity of DNMT3C, suggesting a unique role for this residue in maintaining a proper DNA methylation activity of DNMT3C. Furthermore, this study reveals distinct intramolecular interactions involving the DNMT3C C543-, R548-and E590-corresponding sites: the DNMT3C R548-and E590-corresponding sites form a hydrogen bond in DNMT3C but not in DNMT3A and DNMT3B; the DNMT3C C543- and R548-corresponding sites form a hydrogen bond in DNMT3B but not in DNMT3A and DNMT3C (Fig. 3*D*). Consistently, our DNA methylation analyses revealed that introducing the DNMT3A-converting C543I/E590G and DNMT3B-converting C543N/E590K double mutations, rather than the C543I and C543N single mutations, to DNMT3C substantially shift the DNA methylation activity and specificity of DNMT3C to those of DNMT3A and DNMT3B, respectively. These observations therefore establish that the DNMT3C C543- and E590-corresponding sites coordinately modulate the activity of DNMT3A, DNMT3B and DNMT3C. Intriguingly, our sequence analysis of the DNMT3 family of *de novo* DNA methyltransferases revealed that DNMT3C C543- and E590-corresponding sites are evolutionarily conserved within each DNMT3A, DNMT3B or DNMT3C subfamily but vary between different DNMT3 subfamilies. Together, these findings support a notion that evolutionary covariation of the DNMT3C C543- and E590-corresponding sites may serve to fine-tune the DNA methylation activity and specificity of DNMT3s, opening a new avenue for designing a novel DNA methyltransferase with unique activity and specificity. How the amino acid variations between the DNMT3 family of DNA methyltransferases contribute to their distinct functionalities *in vivo* awaits further investigation.

As with DNMT3B (16), DNMT3C shows a marked methylation activity toward CpA DNA. CpA methylation is the most abundant non-CpG DNA methylation in mammals, occurring in gene bodies, enhancers, promoters, as well as transposons of germ cells, embryonic stem cells, and neural cells (31). An increasing number of studies have linked non-CpG methylation to gene regulation (31, 32, 33). In this context, DNMT3C-mediated non-CpG methylation may provide another mechanism to defend against evolutionarily young transposons. It is also worth mentioning that the ADD domains of DNMT3A and DNMT3B were shown to autoinhibit the activity of DNMT3A/DNMT3B, which can be relieved *via* the interaction between the ADD domain and unmodified histone H3 tail (20, 34, 35). It is conceivable that DNMT3C may adopt a similar regulatory mechanism. However, since the ADD-mediated regulation does not involve a sequence-specific DNA interaction, it may not impact the substrate specificity of DNMT3C substantially at the chromatin level.

Finally, this study provides a molecular basis for the disease-causing *rahu* mutation of DNMT3C, which causes DNA hypomethylation in IAP and L1 transposons, particularly at the 5′-end. A *rahu*-corresponding mutation has not been reported for DNMT3A and DNMT3B. Nevertheless, a three-amino-acid insertion immediately downstream of the DNMT3C E693-corresponding site in DNMT3B has been shown to be associated with the ICF syndrome and perturb protein localization in cells (14, 36). Our structural analysis reveals that DNMT3C E693 is located near to the RD interface, involving interactions stabilizing the oligomerization and the DNA-contact site. Introducing the *rahu* mutation led to impaired oligomerization and reduced DNA-binding activity of the DNMT3C-DNMT3L complex, which consequently abolished the DNA methylation activity of the DNMT3C-DNMT3L complex. Together, this study provides a mechanistic explanation of the functional consequence of the *rahu* mutation, as well as its related mutation in human ICF syndrome.

## Experimental procedures

### Protein expression and purification

A synthetic DNA fragment encoding mouse DNMT3C MTase domain (residues 456–740) and the cDNA encoding the C-terminal domain (residues 178–386) of human DNMT3L (hDNMT3L, NCBI accession NM_175867) or the C-terminal domain (residues 220–415) of mouse DNMT3L (mDNMT3L, NCBI accession NM_1081695) were inserted in tandem in a modified pRSFDuet-1 vector (Novagen Inc), in which the DNA sequence for DNMT3C was preceded by an N-terminal His_6_-SUMO tag and ULP1 (ubiquitin-like protease) cleavage site and the hDNMT3L or mDNMT3L gene was flanked by the NdeI and XhoI cleavage sites. The expression plasmid was transformed in BL21 (DE3) RIL cell strain (Agilent Technologies). The transformed cells were cultured in LB media at 37  °C until the optical density at 600 nm (OD600) reached ∼1.0. The temperature was then lowered to 16  °C, and the cells were induced by 80 μM IPTG (isopropyl β-D-galactoside). The cells continued to grow overnight. Subsequently, the cells were harvested and lysed in a buffer (50 mM Tris-HCl, pH8.0, 1 M NaCl, 10% glycerol, 18 mM Imidazole, 10 μg/ml DNase I, and 1 mM PMSF). The His_6_-SUMO-DNMT3C fusion protein and hDNMT3L/mDNMT3L were co-purified using a Ni-NTA affinity column (GE Healthcare), followed by ion-exchange chromatography on a Heparin HP column (GE Healthcare). After treatment with ULP1 for 2 to 3 h, the His_6_-SUMO tag was removed *via* Ni-NTA affinity chromatography. Finally, the DNMT3C-hDNMT3L or DNMT3C-mDNMT3L complex was purified *via* size-exclusion chromatography on a HiLoad 16/600 Superdex 200 pg column (GE Healthcare) in a buffer containing 25 mM Tris-HCl (pH 7.5), 5% glycerol, 250 mM NaCl, and 5 mM DTT.

For crystallization of the DNMT3C-DNMT3L-DNA complex, the DNA duplex was prepared by annealing of a zebularine-containing palindromic DNA fragment (5′-CATGZGGTCTTAATTAGACCGCATGG-3′; Z: zebularine). Subsequently, the DNA was mixed with the DNMT3C-hDNMT3L tetramer in a 1:2 molar ratio in a buffer containing 50 mM Tris-HCl (pH 8.0), 35% glycerol, and 40 mM DTT at 25 °C. The covalent complex of DNMT3C-hDNMT3L-DNA was first purified using a HiTrap Q HP column (GE Healthcare) pre-equilibrated with 20 mM Tris-HCl, 5% glycerol, 5 mM DTT, and 50 mM NaCl and run in a salt gradient from 50 mM to 1 M NaCl. The protein-DNA complex was further purified on a HiLoad 16/600 Superdex 200 pg column in a buffer containing 20 mM Tris-HCl (pH 8.0), 5% glycerol, 250 mM NaCl and 5 mM DTT. Protein purity was confirmed using SDS-PAGE and the sample was stored at −80  °C before use.

### X-ray crystallography and structure determination

The DNMT3C-mDNMT3L complex shows a reduced solubility than the DNMT3C-hDNMT3L complex. We, therefore, focused on the DNMT3C-hDNMT3L-DNA complex for crystallographic study. The crystallization condition for ∼0.1 mM DNMT3C-hDNMT3L-DNA complex mixed with *S*-adenosyl-homocysteine (SAH) in 1:5 molar ratio was first identified using sparse-matrix screening (Hampton Research Inc). Subsequently, crystals were reproduced and optimized by the hanging drop vapor diffusion method at 4  °C. For optimal crystallization, 1 μl of DNMT3C-hDNMT3L-DNA sample was mixed with 1 μl of the precipitant solution containing 2% v/v Tacsimate TM (pH 8.0), 0.1 M Tris-HCl (pH 8.5), and 16% w/v Polyethylene glycol 3350. To harvest the crystals, the crystals were soaked in mother liquor with an additional 25% ethylene glycol, followed by flash frozen in liquid nitrogen.

The X-ray diffraction data were collected on the Beamline 8.2.2 at the Advanced Light Source, Lawrence Berkeley National Laboratory. The diffraction data were indexed, integrated, and scaled using the HKL3000 program (37), followed by molecular replacement using the PHASER program (38) embedded in the PHENIX software (39), using the structure of human DNMT3B-DNMT3L-DNA complex (PDB 6KDA) as a search model. The structure was refined using COOT (40) and PHENIX software iteratively. The same R-free test set was used throughout the refinement. Structural statistics is listed in Table S1. The structural figures were generated using the Pymol software (https://www.pymol.org/pymol).

### *In vitro* DNA methylation assay

*In vitro* DNA methylation assays were performed following a previously reported protocol (16). In essence, DNA duplexes (GAC)_12_, (AAC)_12,_ and (TAC)_12_ were used for CpG, CpA, and CpT DNA substrates, respectively. For the assays under a single substrate concentration, the experiments were carried out in 20 μl-reaction solution containing 0.75 μM DNA, 0.6 μM DNMT3C-DNMT3L, DNMT3B-DNMT3L or DNMT3A-DNMT3L tetramer, 2.5 μM S-adenosyl-L-[methyl-^3^H]methionine (specific activity 82.3 Ci/mmol, PerkinElmer) in 50 mM Tris-HCl (pH 7.5), 0.05% β-mercaptoethanol, 5% glycerol and 200 μg/ml BSA. The reactions were carried out in triplicate at 37 °C for 10 min unless indicated otherwise, before being quenched by 0.6 μl of 32 mM nonradioactive *S*-adenosyl-methionine (SAM). For the steady-state kinetics assay, the reactions were conducted in a 20-μL solution containing 0.3 μM DNMT3C-DNMT3L C-terminal domains, WT or mutant, various concentrations of CpG DNA (0, 0.0125, 0.025, 0.05, 0.1, 0.2, or 0.35 μM), and 2.5 μM S-adenosyl-L-[methyl-^3^H]methionine (specific activity 82.3 Ci/mmol, PerkinElmer) in 50 mM Tris-HCl (pH 7.5), 0.05% β-mercaptoethanol, 5% glycerol and 200 μg/ml BSA. For measurement of the methylation rate, the reactions were carried out for approximately 0, 6, and 12 min before being quenched by 0.6 μl of 32 mM nonradioactive SAM. The reactions were carried out in triplicate.

Subsequently, 8 μl of each reaction mixture was spot on the Amersham Hybond-XL membrane (GE Healthcare). The membrane was washed sequentially with cold 0.2 M ammonium bicarbonate (pH 8.2) twice, deionized water once, and ethanol once, 15 min for each step. The membrane was air-dried before being transferred to scintillation vials each containing 3 ml of ScintiVerse (Fisher). The ^3^H radioactivity was measured using a Beckman LS6500 counter.

### Electrophoretic mobility shift assay (EMSA)

For the DNMT3C-DNA binding assay, a 12-μL sample contained 1.0 μM (GAC)_12_ DNA duplex mixed with various concentrations (0, 0.5, 1.0, 1.5, and 2 μM) of WT or mutant DNMT3C MTase-mDNMT3L C-terminal domain. The binding mixture was incubated for 30 min on ice in a buffer containing 50 mM Tris-HCl (pH 8.5), 36% glycerol, 8% glucose, 2 mM DTT, 0.1 mg/ml BSA, 100 mM NaCl, and 0.08% Tween 20. Gel electrophoresis was run on an 8% polyacrylamide gel at 110 V using 0.5× TBE (pH 8.3) buffer at 4 °C for 1 h. The gel was stained using SYBR gold stain (Thermo Fisher) for visualization. The band intensity of free DNA in each sample was quantified using ImageJ software (41) and subsequently used for estimation of the ratio of DNMT3C-bound DNA.

### Size-exclusion chromatography

Size-exclusion chromatography analysis was performed on a Superdex 200 increase 10/300 gl column (GE Healthcare). WT and *rahu* DNMT3C protein samples (500 μl of 0.4 mg/ml protein) were injected separately into the column and eluted at a flow rate of 0.5 ml/min in a buffer containing 25 mM Tris-HCl (pH7.5), 5% Glycerol, 250 mM NaCl, and 5 mM DTT buffer. The fractions were collected in 500 μl each. The protein identity was confirmed using SDS-PAGE.

## Data availability

All data needed to evaluate the conclusions in the paper are present in the paper and/or the Supplementary Materials. Coordinates and structure factors for the DNMT3C-DNMT3L-CGG complex have been deposited in the Protein Data Bank under accession code 8TCI.

## Supporting information

This article contains supporting information.

## Conflict of interests

The authors declare that they have no conflicts of interest with the contents of this article.
