# Supplementary material for: The structure of DNA methyltransferase DNMT3C reveals an activity-tuning mechanism for DNA methylation
Source: J Biol Chem. 2024 Aug 2;300(9):107633. doi: 10.1016/j.jbc.2024.107633 (PMC11401227; doi:10.1016/j.jbc.2024.107633)
Supplement: Supplemental data [file mmc1.pdf]

The structure of DNA methyltransferase DNMT3C reveals an activity-tuning mechanism for DNA methylation

Nelli Khudaverdyan[1], Jiuwei Lu[1], Xinyi Chen[1], Genevieve Herle[2], Jikui Song[1,2,#]

[1]Department of Biochemistry, University of California, Riverside, CA 92521, USA

[2]Biophysics program, University of California, Riverside, CA 92521, USA

[#]Correspondence: jikui.song@ucr.edu

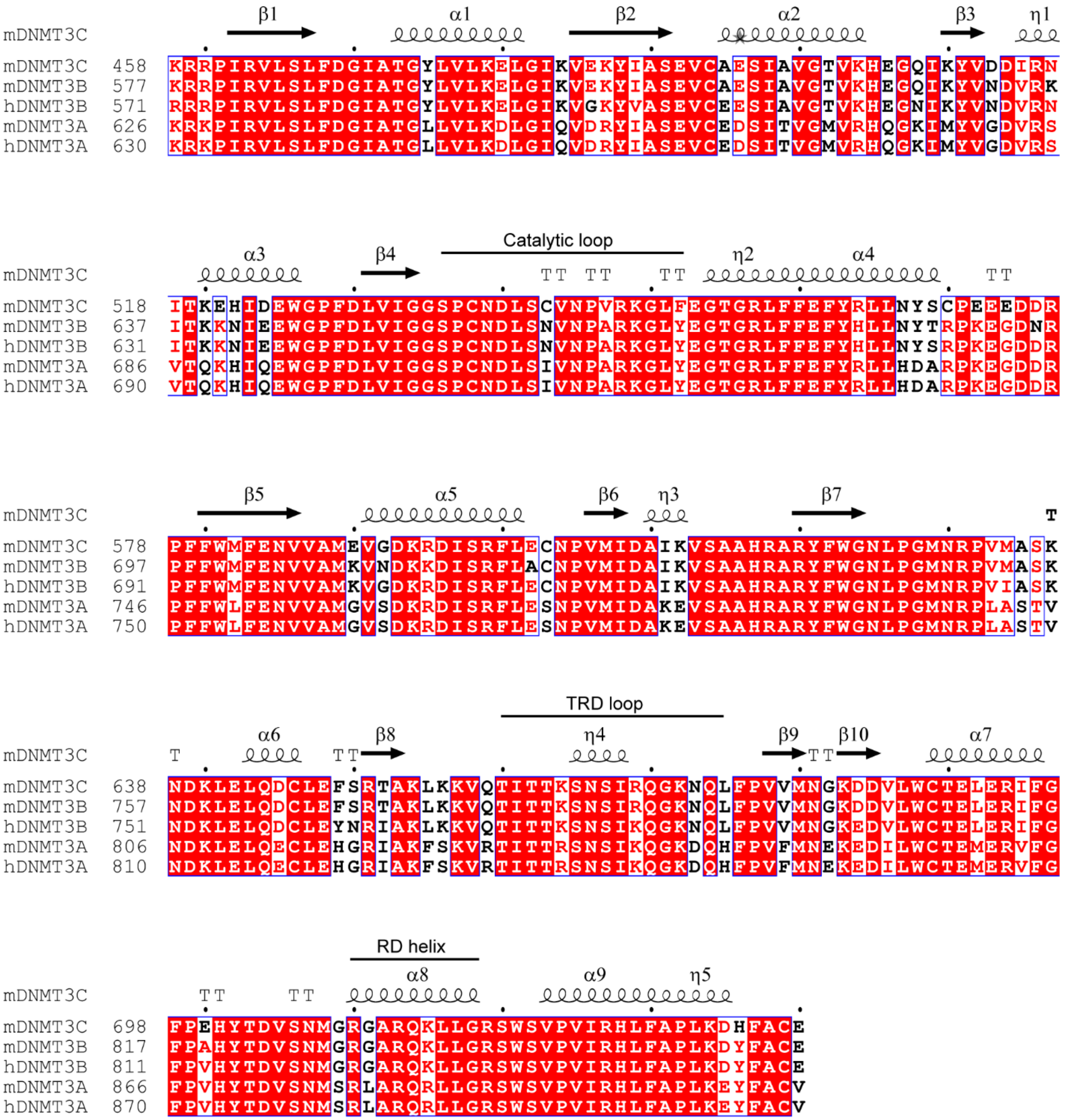

Figure S1. Sequence comparison of DNMT3 proteins. Sequence alignment of the MTase domain of DNMT3A from human (hDNMT3A) and mouse (mDNMT3A), DNMT3B from human (hDNMT3B) and mouse (mDNMT3B), and DNMT3C from mouse (mDNMT3C). Conserved residues are colored white in red background. Similar residues are colored red. The corresponding secondary structures of DNMT3C are marked on top. The DNA-contacting regions (Catalytic loop, TRD loop and RD helix) are also marked on top.

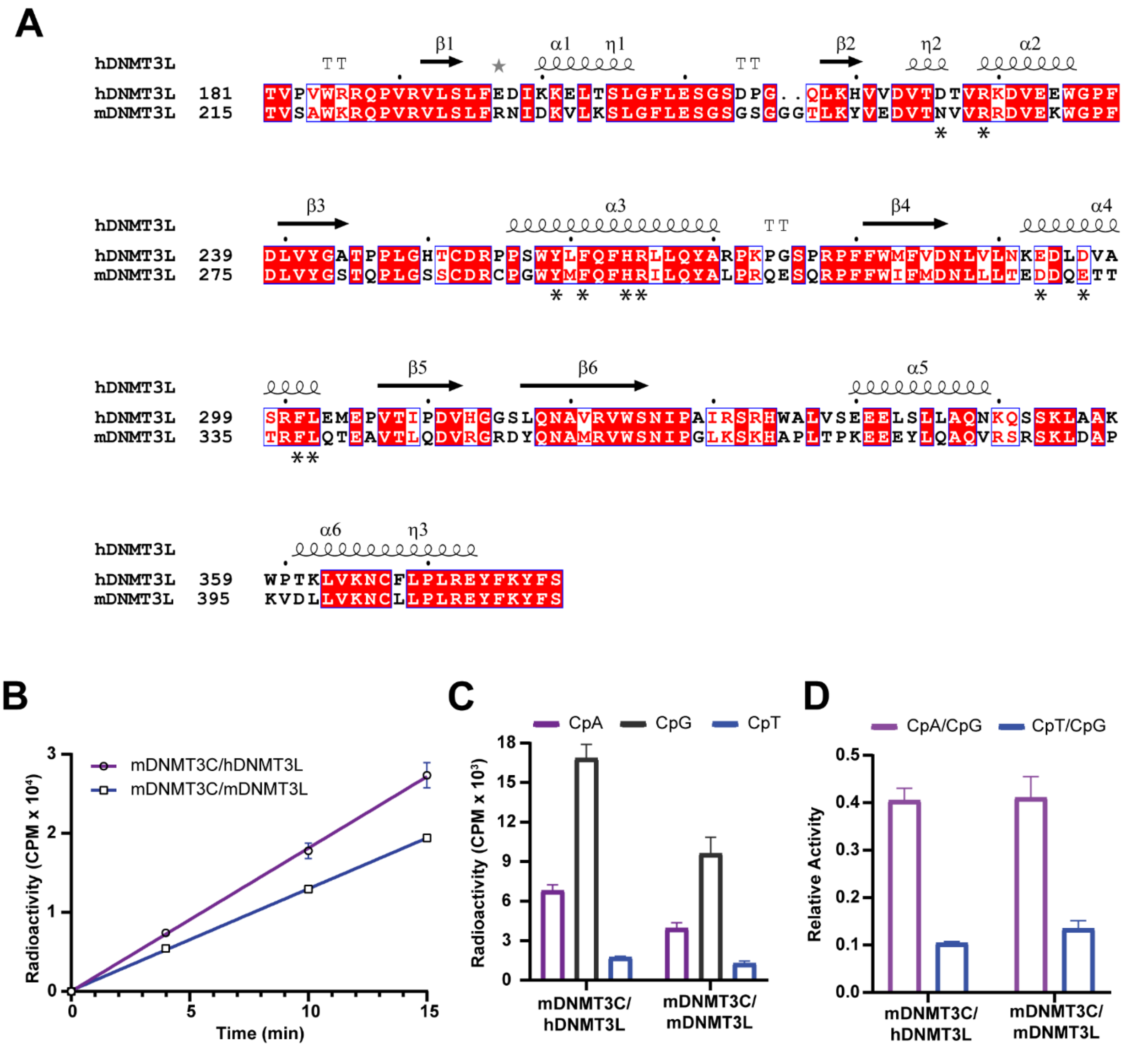

Figure S2. Sequence and enzymatic analysis of DNMT3C-DNMT3L complexes. (A) Sequence alignment of human DNMT3L (hDNMT3L) and mouse DNMT3L (mDNMT3L), marked by the corresponding secondary structures in hDNMT3L on top. Conserved residues are colored white in red background. Similar residues are colored red. Residues in oligomerization interface marked by asterisks. **(B)** *In vitro* DNA methylation kinetics for mDNMT3C-hDNMT3L and mDNMT3C-mDNMT3L complexes on CpG-containing DNA. **(C)** *In vitro* DNA methylation for mDNMT3C-hDNMT3L and mDNMT3C-mDNMT3L complexes on CpA-, CpG- and CpT-containing DNAs after 10-min reaction. (**C)** Relative CpA/CpG and CpT/CpG methylation efficiencies for mDNMT3C-hDNMT3L and mDNMT3C-mDNMT3L complexes.

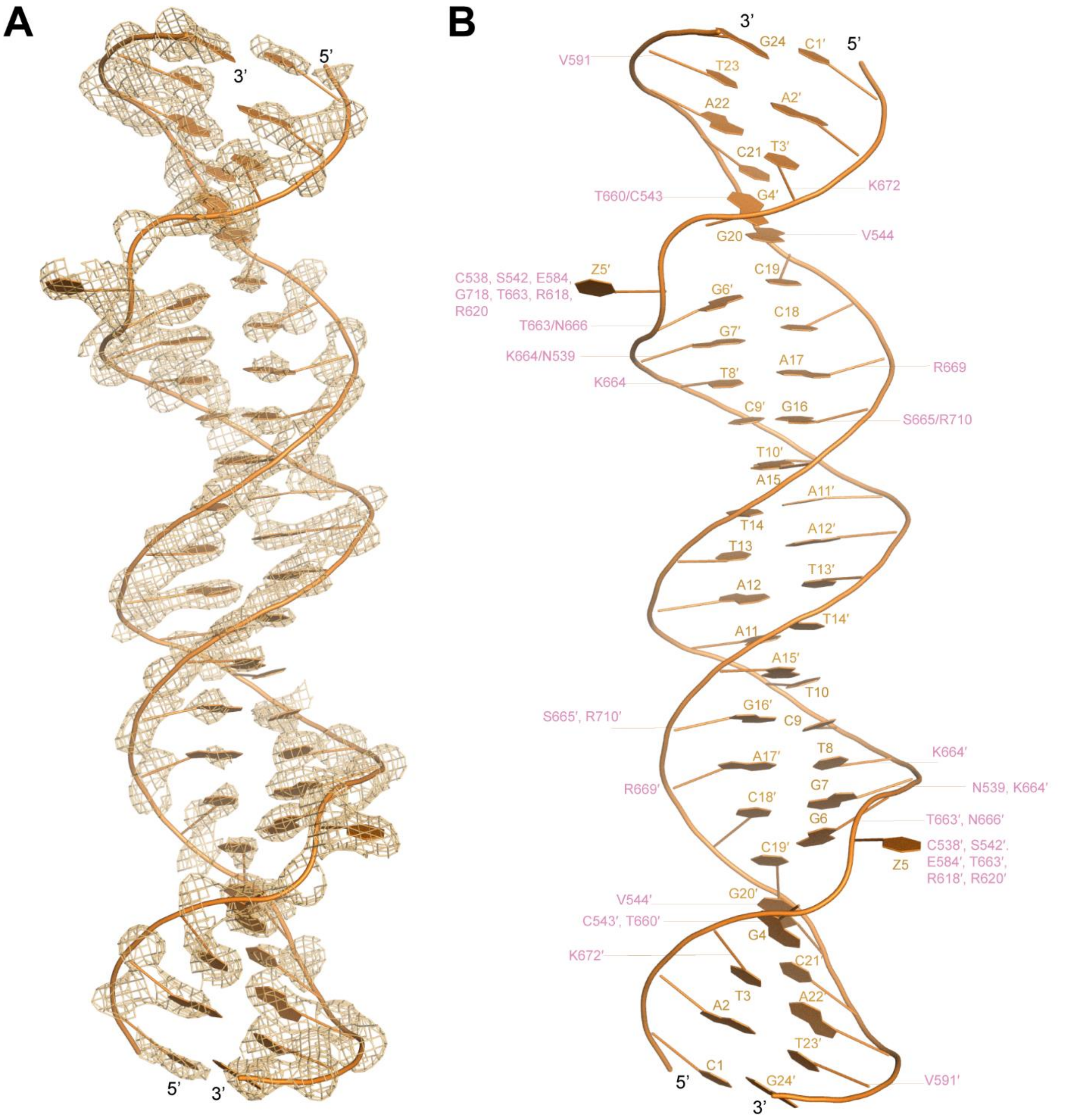

Figure S3. The intermolecular interactions between DNMT3C and CGG DNA. (A) The Fo-Fc omit map of the DNMT3C-bound DNA at 2σ contour level. **(B)** Schematic view of the intermolecular interactions between DNMT3C and DNA. The hydrogen-bonding, electrostatic, or van der Waals contacts are indicated by orange lines between DNMT3C residues (pink) and DNA nucleotides (TV-orange).

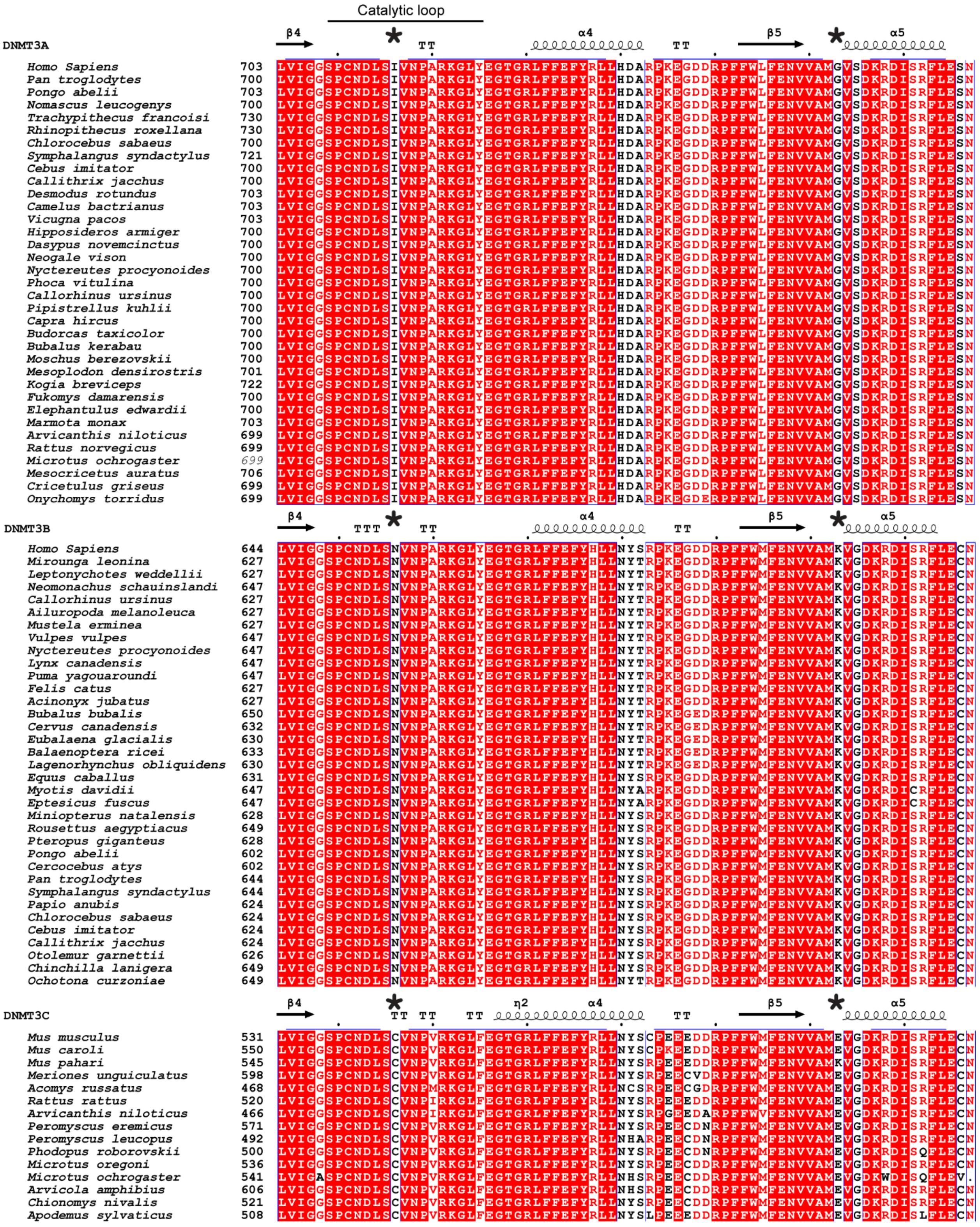

Figure S4. Evolutionary covariation of the DNMT3C C543- and E590-corresponding sites. Sequence alignment of the region surrounding the catalytic loops of DNMT3A,

DNMT3B and DNMT3C from various species. The name of each species is indicated on the left. The secondary structures of human DNMT3A, human DNMT3B and mouse DNMT3B are depicted on the top of the aligned sequences for DNMT3A, DNMT3B and DNMT3C, respectively. The DNMT3C C543- and E590-corresponding sites are marked in black asterisk on top of the aligned sequences. Conserved residues are colored white in red background. Similar residues are colored red.

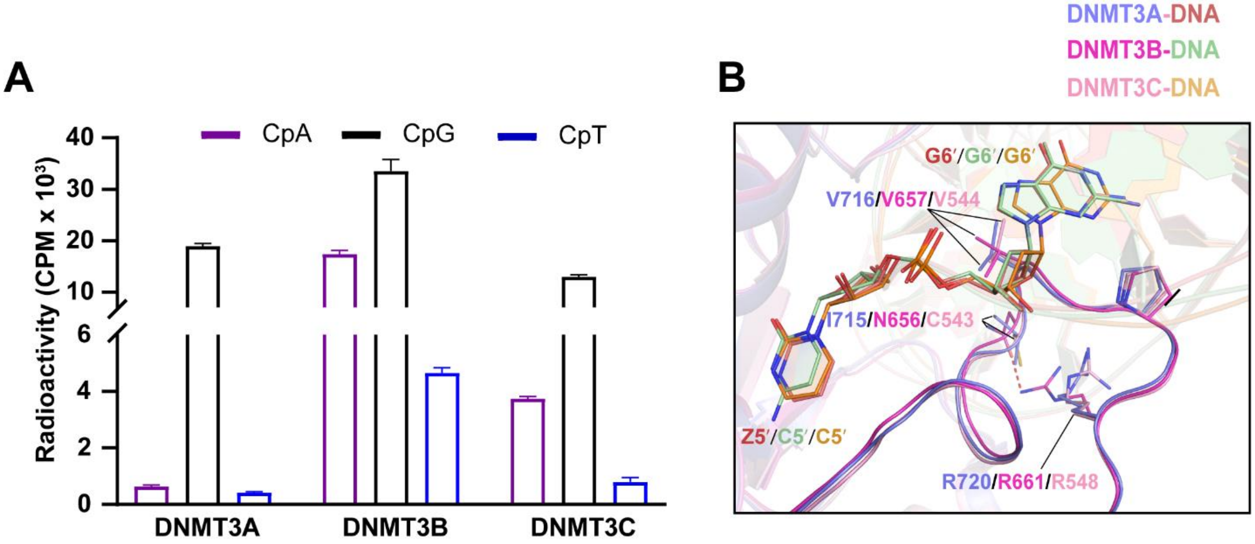

Figure S5. Biochemical and structural analysis of the CpG-specific methylation by DNMT3A, DNMT3B and DNMT3C. (A) *In vitro* DNA methylation analysis of DNMT3A DNMT3B, DNMT3C on CpG, CpA and CpT DNAs. **(B)** Close-up view of overlaid structures of the catalytic loops of DNA-bound DNMT3A (PDB 6W8B), DNMT3B (PDB 6KDA) and DNMT3C, with residues involved in contacts with the CpG guanine (G6′) on the target strand shown in stick representation. The hydrogen bond formed between DNMT3B N656 and R661 is shown as dashed line.

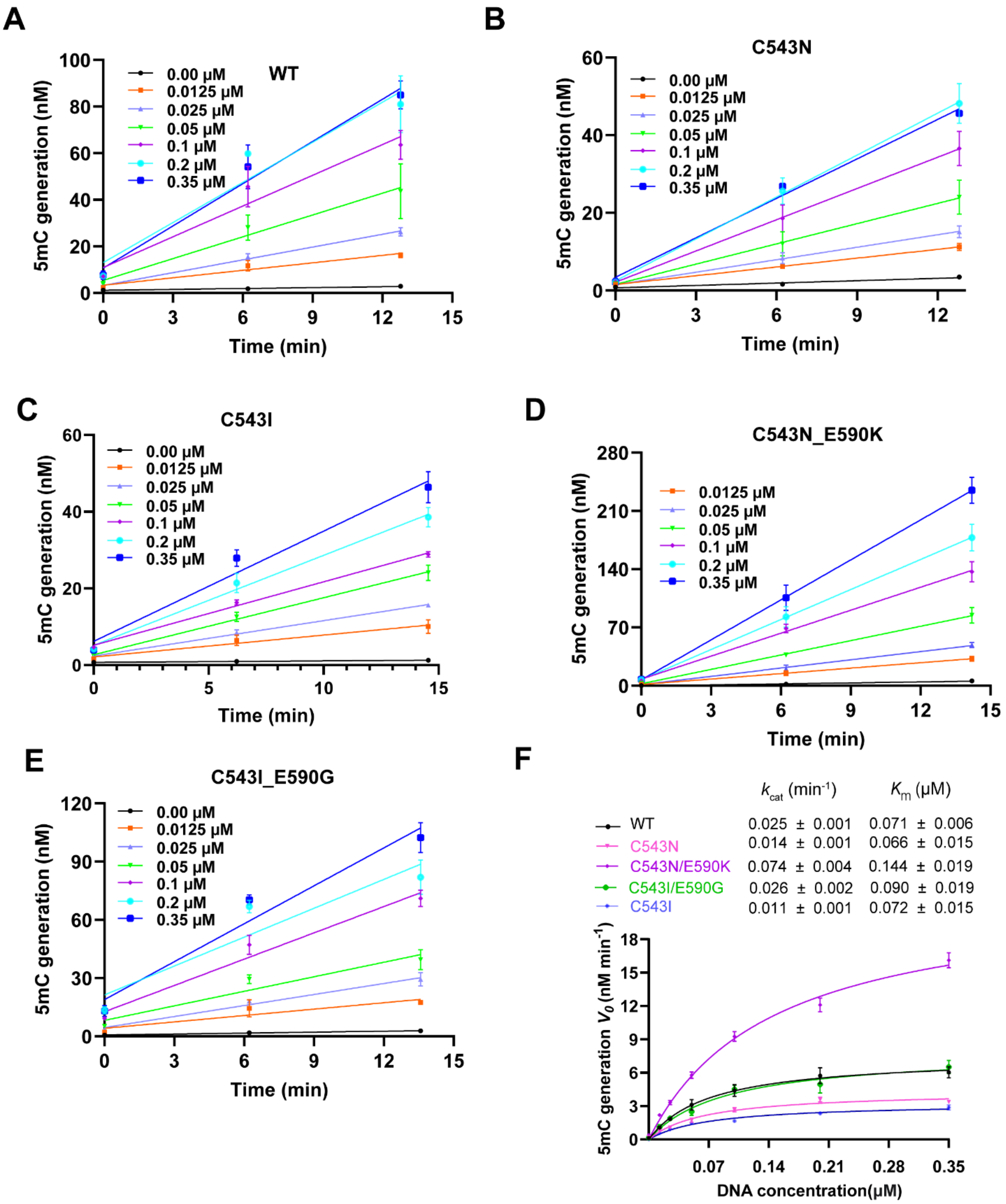

Figure S6. DNA methylation kinetics for DNMT3C-DNMT3L, WT and mutants. (A-E) Substrate concentration-dependent DNA methylation kinetics of WT (A), C543N (B), C543I (C), C543N/E590K (D), and C543I/E590G (E) DNMT3C in the context of DNMT3C-

DNMT3L complex. **(F)** Steady-state enzymatic kinetics of WT and mutant DNMT3C in the context of DNMT3C-DNMT3L complex**.** The k_{cat} and K_m values for each protein are indicated on top.

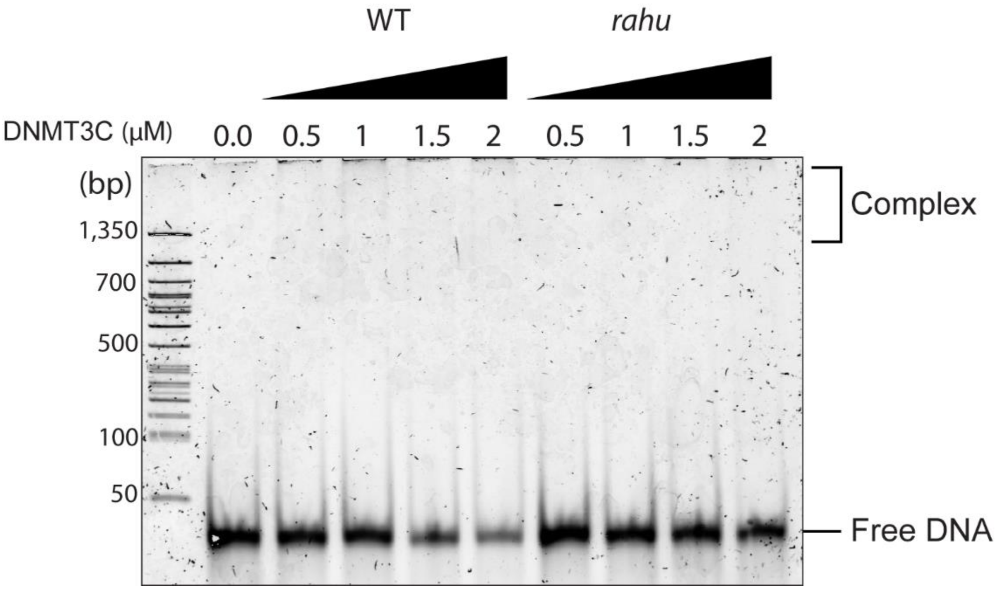

Figure S7. EMSA analysis of the interaction between DNMT3C, WT or *rahu* mutant, and DNA. WT or mutant DNMT3C MTase domain in complex with the C-terminal domain of DNMT3L was used for the DNA binding assay.

Table S1. Crystallographic data collection and refinement statistics of DNMT3C-DNMT3L-DNA complex.

**Data collection**	
Space group	*P 1 21 1*
Cell dimensions	
a, b, c (Å)	63.1, 189.5, 62.9
α, β, γ (°)	90, 90.1, 90
Wavelength	1.0000
Resolution (Å)	50-3.20 (3.31-3.20) [a]
R_{merge}	0.190 (0.999)
$I / \sigma I$	12.1 (1.39)
$CC_{1/2}$	0.930 (0.549)
Completeness (%)	99.5(99.5)
Redundancy	3.4 (3.3)
Total reflections	81785
Unique reflections	23776
**Refinement**	
No. reflections	23704
R_{work} / R_{free} (%)	22.5/25.7
No. atoms	
Protein	7409
DNA	984
SAH	52
B factors (Å^2)	
Protein	73.97
DNA	101.18
SAH	53.56
r.m.s. deviations	
Bond lengths (Å)	0.003
Bond angles (°)	0.57
Ramachandran	
Favored (%)	95.57
Allowed (%)	4.43
Outliers (%)	0

[a]Values in parentheses are for highest-resolution shell. The dataset was collected from a single crystal.
